# The Psychological Impact of COVID-19 on Home Based Primary Care Providers in New York: A Qualitative Study

**DOI:** 10.1093/geroni/igab046.1477

**Published:** 2021-12-17

**Authors:** Emily Franzosa, Sybil Masse, Abraham Brody, Jonathan Ripp, Katherine Ornstein, Alex Federman, Ksenia Gorbenko

**Affiliations:** 1 Icahn School of Medicine at Mount Sinai, Icahn School of Medicine at Mount Sinai, New York, United States; 2 Icahn School of Medicine at Mount Sinai, New York, New York, United States; 3 NYU Hartford Institute for Geriatric Nursing, New York, New York, United States; 4 Mount Sinai Health System, New York, New York, United States

## Abstract

Research on professional burnout during the pandemic has focused on hospital-based health care workers. This study examined the psychological impact of the pandemic on home-based primary care (HBPC) providers. We interviewed 13 participants from six HBPC practices in the New York including medical/clinical directors, program managers, nurse practitioners, and social workers and analyzed the transcripts using inductive qualitative analysis approach. HBPC providers experienced emotional exhaustion and a sense of reduced personal accomplishment. They reported experiencing grief of losing many patients at once and pressure to adapt to changing circumstances quickly. They also reported feeling guilty for failing to protect their patients and reduced confidence in their professional expertise. Strategies to combat burnout included shorter on-call, regular condolence meetings to acknowledge patient deaths, and peer support calls. Our study identifies potential resources to improve the well-being and reduce the risk of burnout among HBPC providers.

